# Parents in Neonatal Pain Management—An International Survey of Parent-Delivered Interventions and Parental Pain Assessment

**DOI:** 10.3390/children11091105

**Published:** 2024-09-09

**Authors:** Alexandra Ullsten, Serdar Beken, Marsha Campbell-Yeo, Giacomo Cavallaro, Nunzia Decembrino, Xavier Durrmeyer, Felipe Garrido, Guðrún Kristjánsdóttir, Abigail Kusi Amponsah, Paola Lago, Helle Haslund-Thomsen, Shalini Ojha, Tarja Pölkki, Monica Riaza Gomez, Jean-Michel Roue, Sinno Simons, Rebeccah Slater, Rikke-Louise Stenkjaer, Sezin Ünal, Gerbrich van den Bosch, Joke Wielenga, Mats Eriksson, on behalf of the ESPR Special Interest Group for Neonatal Pain, PEARL Research Group

**Affiliations:** 1 Faculty of Medicine and Health, School of Health Sciences, Örebro University, S701 82 Örebro, Sweden; alexandra.ullsten@oru.se; 2 Center for Clinical Research and Education, Region Värmland, S651 82 Karlstad, Sweden; 3 Division of Neonatology, Department of Pediatrics, School of Medicine, Acibadem Mehmet Ali Aydinlar University, 34752 Istanbul, Turkey; serdar.beken@acibadem.edu.tr; 4 School of Nursing, Faculty of Health, Dalhousie University, Halifax, NS B3H 4R2, Canada; 5 Neonatal-Perinatal Division, Department of Pediatrics, IWK Health Centre, Halifax, NS B3H 4R2, Canada; 6 Neonatal Intensive Care Unit, Fondazione IRCCS Ca’ Granda Ospedale Maggiore Policlinico, 20122 Milan, Italy; giacomo.cavallaro@policlinico.mi.it; 7 Neonatal Intensive Care Unit, AOU Policlinico G. Rodolico San Marco, 95123 Catania, Italy; 8 Neonatal Intensive Care Unit, Centre Hospitalier Intercommunal de Créteil, 94000 Créteil, France; 9 GRC CARMAS, IMRB, Faculté de Santé de Créteil, Université Paris Est Créteil, 94000 Créteil, France; 10 Department of Pediatrics, Clínica Universidad de Navarra, 28027 Madrid, Spain; 11 Faculty of Nursing and Midwifery, School of Health Sciences, University of Iceland, IS-101 Reykjavik, Iceland; gkrist@hi.is; 12 Landspitali University Hospital, IS-101 Reykjavik, Iceland; 13 Department of Nursing, Faculty of Allied Health Sciences, College of Health Sciences, Kwame Nkrumah University of Science and Technology, University Post Office, KNUST, Kumasi, Ghana; 14 Department of Nursing Science, Faculty of Medicine, University of Turku, FI-20014 Turku, Finland; 15 NICU, Department of Critical Care, Cà Foncello Regional Hospital, 31100 Treviso, Italy; paola.lago@aulss2.veneto.it; 16 Clinical Nursing Research Unit, Aalborg University Hospital, 9000 Aalborg, Denmark; 17 Department of Paediatrics, Aalborg University Hospital, 9000 Aalborg, Denmark; 18 Department of Clinical Medicine, Aalborg University, 9000 Aalborg, Denmark; 19 Centre for Perinatal Research, School of Medicine, University of Nottingham, Nottingham NG7 2RD, UK; shalini.ojha@nottingham.ac.uk; 20 Research Unit of Health Sciences and Technology, Faculty of Medicine, University of Oulu, FI-90014 Oulu, Finland; tarja.polkki@oulu.fi; 21 Medical Research Center Oulu, Oulu University Hospital, University of Oulu, FI-90014 Oulu, Finland; 22 Clínica Universidad de Navarra, 31008 Pamplona, Spain; 23 Department of Neonatal Medicine, University Hospital of Brest, 29200 Brest, France; 24 Department of Neonatal and Pediatric Intensive Care, Division of Neonatology, Erasmus UMC–Sophia Dr. Molewaterplein 40, 3015 GD Rotterdam, The Netherlands; 25 Department of Paediatrics, University of Oxford, Oxford OX1 2JD, UK; rebeccah.slater@paediatrics.ox.ac.uk; 26 Department of Neonatology, Copenhagen University Hospital, Rigshospitalet, 2100 Copenhagen, Denmark; rikke.louise.stenkjaer@regionh.dk; 27 Division of Neonatology, Baskent University Faculty of Medicine, 06790 Ankara, Turkey; sezinunal@baskent.edu.tr; 28 Emma Children’s Hospital, 1105 AZ Amsterdam, The Netherlands

**Keywords:** pain, parents, newborn infants, neonatal pain, parent-delivered pain management, skin-to-skin contact, breastfeeding, infant-directed singing

## Abstract

Background: While parent-delivered pain management has been demonstrated to effectively reduce neonatal procedural pain responses, little is known about to what extent it is utilized. Our aim was to explore the utilization of parents in neonatal pain management and investigate whether local guidelines promote parent-delivered interventions. Methods: A web-based survey was distributed to neonatal units worldwide. Results: The majority of the 303 responding neonatal intensive care units (NICUs) from 44 countries were situated in high-income countries from Europe and Central Asia. Of the responding units, 67% had local guidelines about neonatal pain management, and of these, 40% answered that parental involvement was recommended, 27% answered that the role of parents in pain management was mentioned as optional, and 32% responded that it was not mentioned in the guidelines. According to the free-text responses, parent-delivered interventions of skin-to-skin contact, breastfeeding, and parental live singing were the most frequently performed in the NICUs. Of the responding units, 65% answered that parents performed some form of pain management regularly or always. Conclusions: There appears to be some practice uptake of parent-delivered pain management to reduce neonatal pain in high-income countries. Additional incorporation of these interventions into NICU pain guidelines is needed, as well as a better understanding of the use of parent-delivered pain management in low- and middle-income countries.

## 1. Introduction

Parent-delivered procedural pain management has been demonstrated to provide effective pain relief in both healthy and sick full-term and preterm infants [1]. When parents are encouraged, well informed, and prepared, parents are effective mediators of pain relief. As such, parents are an essential component of optimal evidence-based, family-centered neonatal pain management [2]. Moreover, most parents wish to take an active role in helping their infant manage procedural pain [3,4,5], which can make them feel useful, empowered, and reassured [5,6,7]. Skin-to-skin contact, breastfeeding, and parental musical vocalizations are some examples of pain-reducing interventions that allow parents to reduce their infant’s pain and at the same time lower their anxiety and develop their parenting skills [5,8]. Parents can also participate in delivering, e.g., facilitated tucking, non-nutritive sucking, or holding/swaddling [9,10]. Another way that parents can be actively engaged in pain management is assisting with pain assessments. While there are a growing number of pain studies from around the world supporting the effectiveness of parent-delivered pain management, less is known regarding parent-assisted neonatal pain assessments [11].

There is significant global variability in analgosedation pain management practices in infants [12]. We have reason to believe that similar worldwide variabilities in neonatal practices and professionals’ attitudes apply to parent-delivered pain-reducing interventions. While new guidelines on family-centered or family-integrated care are revised regularly, many lack recommendations for infant pain management, and consequently, few guidelines include recommendations regarding parent-delivered interventions, although there are notable exceptions [13].

There are many advantages of involving parents in pain management, to the benefit not only of the infant and parent but also in the interest of health care, i.e., by improving interprofessional collaboration and family-centered care [14,15]. There is a need for more knowledge related to what extent parents are accepted as active partners in infant pain management in NICUs globally and whether local guidelines promote parent-delivered interventions and parental pain assessment. Specifically, we are interested in whether parents deliver pain management in clinical settings on a day-to-day basis, and if so, how they are actively involved, what kind of pain management parents frequently provide, and during what kind of painful situations.

Therefore, the aim of this study was to explore the role of parents in neonatal pain management. Its specific aims were to investigate to what extent parents are involved in pain management in NICUs throughout the world and whether local guidelines promote parent-delivered interventions and parental pain assessments in neonatal pain management.

## 2. Materials and Methods

This study was conducted as a cross-sectional global web survey with 35 pre-defined multiple-choice answers and the option to provide free-text answers in all sections.

### 2.1. The Construction of the Survey

The survey was developed during discussions within the Pain in Early Life (PEARL) research group and the European Society for Paediatric Research (ESPR) Special Interest Group (SIG) for Neonatal Pain (online Supplementary Material S1 and S2) and consisted of questions about (a) the responding NICUs, (b) pain guidelines, (c) parent-delivered interventions, (d) parental pain assessments, and (e) the attitudes of the staff towards parental involvement in neonatal care (online Supplementary Material S3). The survey explored the conditions at the unit level, i.e., not at the individual level, and each NICU responded once to the survey. The survey was directed to the person on the unit with the most knowledge about pain management. Depending on how the NICUs were organized, the respondent included neonatologists, NICU nurses, unit managers, etc.

### 2.2. Distribution

The web-based survey was made available in English (online Supplementary Material S3). Information about the survey and a link to the survey were distributed via several clinical and parent networks, professional organizations, and personal contacts. The survey was open for responses from June 2023 to the end of October 2023. Ethical approval was not applicable to this survey since no personal data were collected.

### 2.3. Statistical Analysis

The results are presented as numbers and percentages or median and quartiles. To search for units with the same pattern of parental participation, we performed a Latent Class Analysis (LCA) [16]. As manifest indicator variables, we chose all variables that described how much parents participated in pain management and pain assessments. Then, we conducted sequence modeling with 10 iterations, starting with 1 class, until the lowest Bayesian Information Criterion (BIC = 9411.843) was reached, indicating that a 3-class model had the best fit [16].

Comparative analyses were made using chi-square tests for AAP level of neonatal care [17], sociodemographic index (SDI) [18], and the three LCA classes.

### 2.4. Quantitative Content Analysis

Each section of the survey included free-text questions to provide the respondents with the opportunity to explain a previous answer to the closed-ended survey questions and share additional information and opinions on various topics.

The authors A.U. and M.E. manually extracted all the text responses from the survey and organized them into a main Excel data file, sorted by country and hospital unit. Prior to the analysis, some of the free-text responses required translation from their original language into English by native-language-speaking health care professionals with experience in neonatal care. The dataset included all the free-text responses, analyzed using quantitative content analysis [19]. A coding scheme was developed inductively from the data guided by the research questions and the concepts of the study. As part of the quantitative content analysis, a frequency count was performed by counting the number of responses mentioning a particular content or topic. The results are illustrated with representative quotes related to each topic.

## 3. Results

### 3.1. The Responding Units

**Figure 1:**
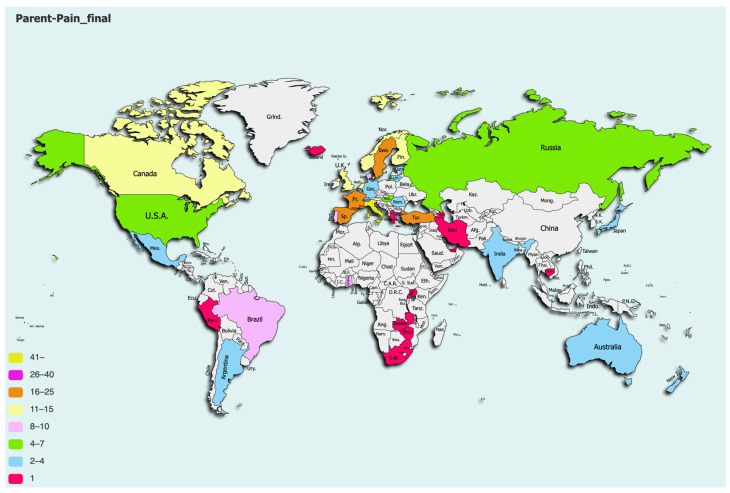
Responding units per country.

The survey was completed by 320 respondents. Only one respondent per NICU was included, so after removing duplicate answers from the same units, the final dataset consisted of answers from 303 units in 44 different countries (Figure 1). The majority (79.2%) of the responding units were situated in high-income countries from Europe and Central Asia, with a median SDI of 0.805 (q1–q3: 0.767–0.872). See Table 1 for a description of the units. The surveys were primarily answered by neonatologists (63%), followed by nurses or nurse practitioners (27%).

### 3.2. Parent-Delivered Interventions in the Guidelines and Protocols

Of the responding units, 67% reported that they had local guidelines for pain management. When asked whether the role of parents in pain management was included in the guidelines, 32% answered that it was not mentioned, 27% that it was mentioned as an option, and 40% that parental involvement was recommended. There were no significant differences depending on level of care, the SDI, or LCA class.

According to the free-text responses, skin-to-skin contact (SSC), breastfeeding, and parental live singing were most frequently mentioned in the local guidelines. However, most of the respondents stated that there were no written recommendations for parent-delivered pain management, but their NICU nonetheless used these strategies or encouraged parent-delivered interventions but not in a systematic way:

“Some of these parent-led methods are commonly practiced, but they are not mentioned in our written guidelines”.

“Live singing is encouraged but not in the written recommendations”.

If combined parent-delivered pain-relieving interventions were mentioned or recommended in the guidelines or protocols, the combination of breastfeeding and SSC was the most recommended according to the free-text responses. Other combinations that were mentioned in the guidelines according to the answers were combined breastfeeding, SSC, and parental live singing/humming; combined SSC and parental live singing/humming; combined breastfeeding and parental live singing/humming; and parental singing in combination with sensorial saturation, massage, and touch.

### 3.3. Parent-Delivered Interventions Performed

Of the responding units, 65.1% answered that parents performed some form of pain management on a regular basis or always. There were no significant differences depending on level of care or country SDI, whereas only 27.6% in the low-participation units (see definitions of the LCA classes below) incorporated pain management by parents on a regular basis or always (*p* < 0.001). Comforting touching by parents was the most frequent single intervention, used by 77.0% of the units, followed by SSC (71.2%), containment (63.2%), non-nutritive sucking by parents (59.0%), and breastfeeding (57.1%). Of the responding units, 42.9% answered that combined parent-delivered interventions are mentioned in their guidelines or local protocols, and 36.3% of the units stated that they use combined interventions in pain management on a regular basis or always.

**Figure 2:**
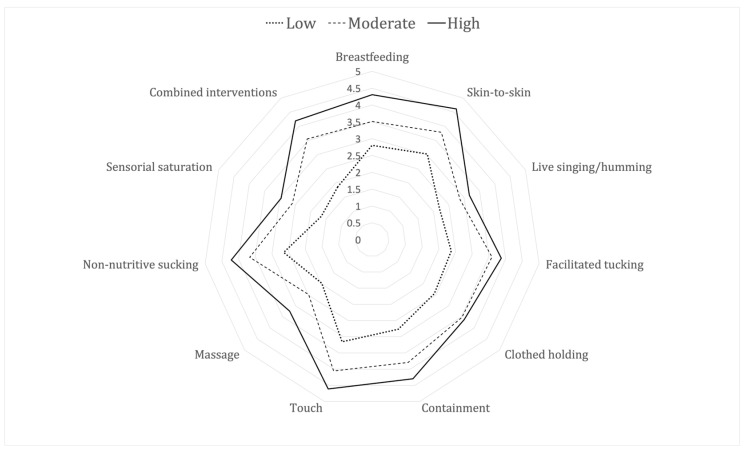
Radar diagram showing three levels of parental pain management: units with low, moderate, or high participation, where 1 is never and 5 is always.

The following indicator variables were included in the Latent Class Analysis: parents performing pain relief in general, performing any of breastfeeding, SSC, live singing, facilitated tucking, holding, swaddling, touching, massaging, providing non-nutritive sucking or sensorial saturation, or combinations of any of these interventions or performing pain assessments. The best fit of the LCA suggested three classes with different patterns of parental participation in pain management: low, moderate, and high levels of participation. Figure 2 demonstrates how often the parents performed the different interventions in the three classes of units.

Table 2 demonstrates the properties of the units belonging to the three different LCA-classes. There was a tendency towards some regional differences, but due to the low numbers, no significant differences were seen between the classes in terms of any of the analyzed aspects.

**Figure 3:**
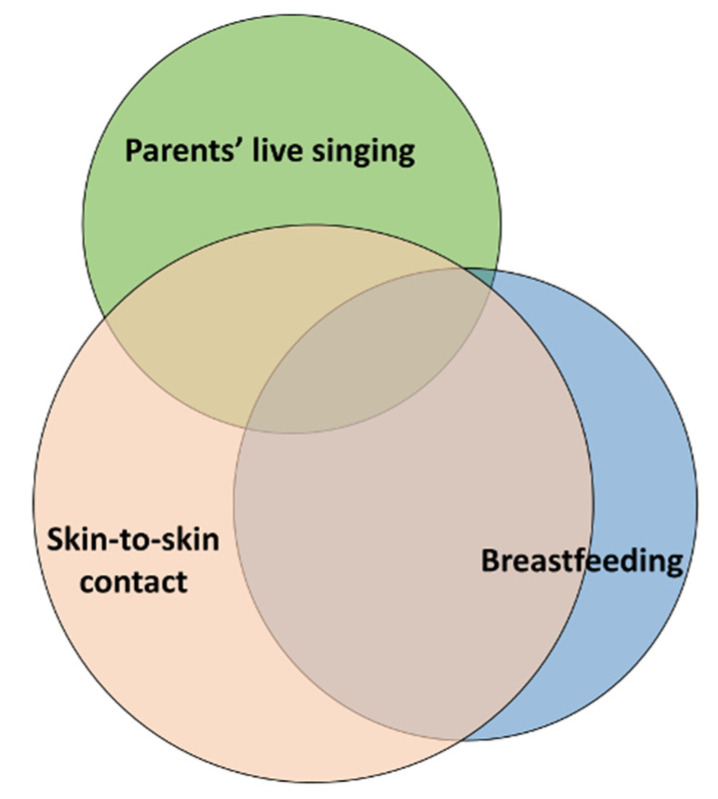
The most frequently combined parent-delivered pain-relieving interventions performed in the NICUs according to the free-text responses in the survey. Overlapping circles indicate units that reported more than one of these combinations.

As illustrated in Figure 3, in the free-text responses, the respondents commented on the parent-delivered combinations that were most frequently performed in the NICUs. The combination most mentioned was skin-to-skin contact and breastfeeding (33 units), followed by skin-to-skin contact and parental live singing/humming (15 units). Combined breastfeeding and parental live singing were mentioned by six units, and the combination of all three parent-delivered interventions was also mentioned by six units.

### 3.4. Pain Assessment Performed by Parents

Twenty-one per cent of the responding units reported that pain assessment by parents was mentioned in the guidelines. There were no significant differences depending on level of care or SDI, but the low-performing units reported a prevalence of 17.1% (*p* < 0.001).

When asked whether parents performed pain assessments, 12.9% of the units reported that they did on a regular basis or always. Again, there were no significant differences depending on level of care or SDI, whereas the proportion at the high-performing units was 32.4% (*p* < 0.001).

In the free-text responses, the respondents commented on in what situations parents performed pain assessments and whether they assessed pain, and if so, with what scale. The most frequent situations mentioned in the responses were blood-sampling procedures, immunization, airway suctioning, tape removal, the insertion of IV cannulas, after surgery, and during mechanical ventilation. Parents were more involved in pain assessments if they stayed regularly in the units. In some units, staff had started to informally implement parental pain assessments, while other units stated that pain assessment is the nurses’ and physicians’ responsibility:

“Parents are not instructed on how to assess pain formally using a validated pain scale. We do tell them how to recognize signs of pain and encourage parents to voice their concerns about pain levels but not using a pain score based on a validated pain scale”.

While a few pain scales were referred to as used in the units (EDIN, PIPP, ALPS Neo, COMFORT Neo, N-PASS, and FLACC), most of the respondents answered that parents do not use a pain scale. Instead, they verbally communicate their observations to the staff:

“I think a pain scale should first be validated for use by parents”.

“We use the ALPS for parents to help them to read their babies signals. Mostly used for parents to premature babies. Even when ALPS is not used, we encourage parents to tell us if they see signs of pain or discomfort”.

### 3.5. Health Care Professionals’ Attitudes towards Parent-Delivered Interventions

**Figure 4:**
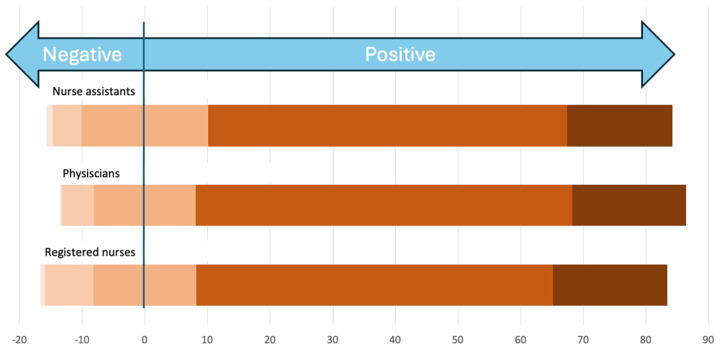
The perceived opinion of nurse assistants, physicians, and registered nurses and their perception and attitude towards parental-delivered pain-relieving interventions, from very negative (left, light peach color) to very positive (right, dark brown color).

The units were asked about the opinions of the staff towards parents participating in pain management. All three groups of professionals were reported to mainly feel positive about involving parents (Figure 4). There were no significant differences depending on the level of care. The nurses were more positive in high–middle- and high-SDI countries (*p* = 0.031), as were physicians (*p* = 0.012). All professions from moderate- and high-performing units were more positive (*p* > 0.001).

In the free-form text responses, the health care professionals expressed some downsides of including parent-delivered interventions and having parents present during painful procedures. There were mixed attitudes among nurses, physicians, and allied professionals towards letting parents provide pain management, especially during more invasive procedures:

“Still, many nurses prefer that parents stay away during pain maneuvers; I mean invasive ones; while all of them are very positive for parents’ participation in pain relief during simple procedures such as heel puncture”.

The responses also supported the idea that less experienced health care professionals often involve parents less:

“Especially young doctors with less experience feel uncomfortable being watched doing blood withdrawals and venous punctures”.

Stress, time constraints, high workloads, and a lack of a unified approach among the staff were expressed as barriers to including parents in pain management, as were structural and organizational challenges:

“We know it is a subject that needs to be discussed, but it always becomes second to other subjects for example ventilation, infection, etc.”.

“Nurses believe that parents in some way disturb the nursing care routine and can transmit infections”.

“Even if I would like to have parents in NICU all day, the hospital Director doesn’t agree”.

The respondents also commented that parents’ access to their infants during painful situations might depend on the parents’ overall access to and presence in the unit, as well as on the pain care culture of each NICU:

“Parents could take part more in pain relief if they were present more with the baby in the neonatal intensive care unit. Only a few parents are on the unit around the clock”.

“Culturally, parents and staff are not comfortable with each other during procedures. I mean staff are not comfortable doing procedures in the presence of parents and parents also don’t want to be around when procedures are being done on baby”.

### 3.6. Parents’ Attitudes towards Parent-Delivered Pain Management

The units were asked how the parents would react if they were asked to participate in pain management. Of all the respondents, 91.7% declared that the parents would feel positive or very positive about it. There were no significant differences depending on level of care or country SDI. For moderate-performing units, the proportion was 94%, and for high-performing units, it was 97.2% (*p* = 0.002).

The health care professionals expressed in their free-form text responses that they often met anxious parents who were reluctant to provide their infants with pain management but that these parents gained confidence in engaging within a short time:

“In the beginning the parents are worried about their ability to care for the baby; after a couple of days, they are much more confident, and they feel ready to care”.

“The reaction of the parents depends on their education and socioeconomic level”.

### 3.7. Information Given to Parents

Less than half of the responding units provided information to parents on parent-delivered pain management. A total of 5% of the units used an app to inform parents about pain management, 8.7% used the internet, and 35.1% used booklets or brochures.

**Figure 5:**
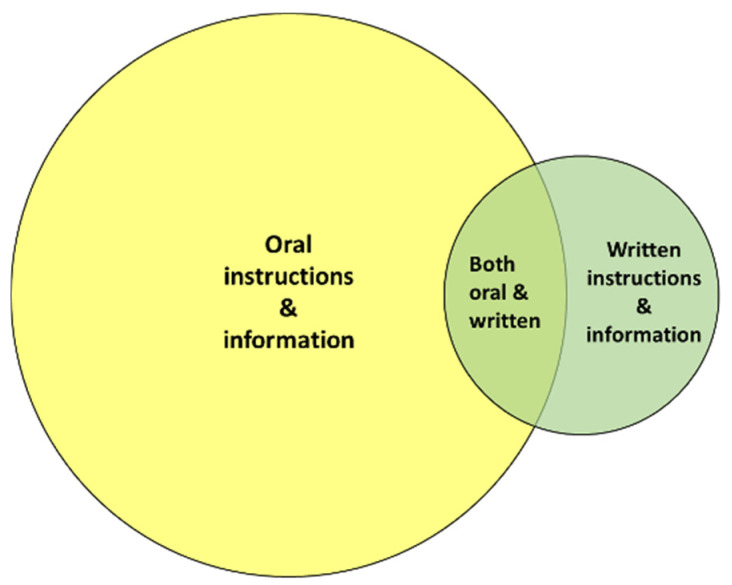
Health care professionals generally transfer knowledge about parent-delivered pain-relieving interventions to parents orally.

As shown in Figure 5, according to the free-form text responses, health care professionals predominantly inform and instruct parents about parent-delivered pain-relieving interventions orally. Written instructions and information with booklets, posters, and educational videos are less used:

“We have a “Parents guide to the NICU” with information in all family rooms that include information about parent-delivered-relieving interventions”.

“QR code at the unit’s entrance to download Linda Frank’s French version of Comforting Your Baby in the NICU”.

## 4. Discussion

This is the first web-based survey distributed to NICUs globally exploring the utilization of parent-delivered interventions for neonatal pain management and pain assessments from health care professionals’ perspectives. Most of the responding units have local guidelines on neonatal pain management, but only a minority of these guidelines recommend parent-delivered pain management and parental pain assessment. Despite concerns about stress, time constraints, high workloads, etc., the majority of the health care professionals answering this survey were positive about including parents in management of their infants’ pain and assessing pain. It is promising that as many as 65.1% of the respondents stated that the parents performed some kind of pain management on a regular basis or always, and the free-text answers confirmed that despite then having no written recommendations for parent-delivered pain management, many NICUs around the world nonetheless use these interventions and encourage parents to provide pain management. Detailed guidelines on parent-delivered interventions during painful procedures could further support both health care professionals and parents in these positive endeavors [13]. Importantly, the most reported single parent-delivered intervention was comforting touching by parents, which would not be considered an effective intervention for reducing pain. This highlights the importance of the education and information provided to both health care providers and families regarding the most effective parent-delivered interventions being evidence-based.

The most frequently cited combined parent-delivered pain management techniques performed in the NICUs, according to the free-text responses in the survey, were skin-to-skin contact and breastfeeding, followed by skin-to-skin contact and parental live singing/humming. Skin-to-skin contact [20] and breastfeeding [21] are among the most studied parental pain-relieving interventions [9], being easy and safe to implement. Recent research shows that maternal live singing is feasible and beneficial for both mothers and their preterm infants in reducing procedural pain in preterm infants, as well as decreasing mothers’ anxiety levels and increasing oxytocin levels in both infants and mothers [22,23]. Research also recommends that health care professionals support parents live singing when infants are undergoing painful procedures [24]. Many of the respondents commented in their free-text answers that their NICUs were about to update, or recently had updated, their local guidelines to also include and recommend parent-delivered pain management tactics such as skin-to-skin contact, in combination with, for example, breastfeeding or parental live singing. Hopefully, this survey will inspire these NICUs to intensify their work.

However, it was evident in the survey that including parents in pain management and pain assessment is dependent on individual initiatives and is seldom formalized or initiated consistently. This is a major area for improvement globally. Reflective and progressive management and leadership, pain management guidelines, protocols which include parent-delivered pain-reducing interventions, parental pain assessment policies, and plans for parental education are known factors that could facilitate the implementation of parent-delivered pain management [2].

According to the survey results, the same applies to knowledge-sharing, which is not formalized in the NICUs either. Research has previously shown that many parents remain unaware of their capacity to provide their infants with pain relief, which contributes to poor practice [2]. Formal (e.g., from health care professionals) and informal (e.g., from peer groups) written and oral information, parent-targeted education, and online e-health resources advising parents on the importance of their role in pain management are key to clinical change leading to the successful involvement of parents in their infants’ pain management and better pain care [2].

The responses in the survey suggested that the respondents perceived that health care professionals being less experienced and more invasive procedures being performed equated to less involvement of parents. Health care professionals’ age, gender, experience, status, education level, expertise, and confidence may contribute to or hamper parent-delivered pain management [25,26,27]. The negative free-text responses regarding these barriers highlight significant attitudes and beliefs in health care providers that continue to hamper the inclusion of parents as partners in pain management. Health care professionals need education and training that supports parental involvement, and the development of professional knowledge consists of developing guidelines on parent-delivered pain management, the continuous presentation of consolidated new research findings, regular evaluation of pain management outcomes, and periodic education for new staff [2].

The physical presence of parents in an NICU is evidently an important prerequisite for parent-delivered pain management. The units from Nordic countries and Canada that answered the survey seemed to have well-developed infrastructures and supportive pain care cultures for parents’ participation in pain management, as also seen in previous studies [28,29]. However, most of the NICUs that participated in the survey are unable to provide a family-centered environment with family-friendly facilities and single rooms for families and described in their free-form text answers how they only provided the opportunity for parents to be present during the daytime, offering the parents chairs or recliners for SSC. The financial burden for parents of having an infant in neonatal care, limited physical space, and a lack of family rooms within NICUs, as well as inadequate social support systems, are serious threats to parents’ ability to provide pain relief [2], and such barriers are important to address when striving to improve neonatal pain management.

### Strengths and Limitations

This survey reached 303 unique NICUs in 44 countries and is the first survey to investigate to what extent parent-delivered interventions are used in clinical practice. A significant limitation of the survey was that only one person per unit provided a response. This design introduces a high risk of bias in the perceived utilization of parent-delivered pain management and perceived barriers and assumptions regarding health care professionals’ beliefs. We have previously used this design when exploring unit policies and routines [29], but there is a risk that this may have contributed to a more positive representation of the utilization of parent-delivered pain management than demonstrated in previous studies, e.g., from Finland and Estonia [30,31]. Nevertheless, our survey has provided a first insight into parent-delivered pain management, policies, and health care providers’ attitudes and beliefs across multiple geographic settings and levels of patient acuity. While the focus was clinical practice settings, another limitation is that the survey was completed only by health care providers without parental input. Future surveys should provide the opportunity for parents’ voices to be included.

Even though the intentions of our survey were ambitious, we did not manage to reach a sufficient number of NICUs in low-income and low-tech settings. This highlights the challenges and limitations of distributing global surveys via networks and professional organizations which are not represented in all countries. The Latent Class Analysis could identify three levels of parental participation in pain management. We saw some interesting trends when looking at the properties of the units in the three classes, but due to low numbers, we could not show any significant differences.

Among the health care professionals that answered this survey, their attitudes towards parent-delivered interventions were mainly positive, and the proportion of units that reported that they included parents in pain management was surprisingly large. This may imply that those who are negative or uninterested did not take part in the survey. This survey may lack answers from NICUs that are more negative towards parent-delivered pain management.

The survey having only been distributed in the English language may have also limited responses from health care professionals with no or little knowledge in a second language.

## 5. Conclusions

Parent-delivered pain management is a growing concept across NICUs and health care globally. The results from this global web survey support the notion that parent-delivered interventions may currently be used in clinical practice on a day-to-day basis in 65.1% of the responding units but may only be recommended in the local guidelines in 40% of them. Developing guidelines on parent-delivered pain management should therefore be a global priority in neonatal care to drive and accelerate clinical change and provide better pain management for infants and their parents. Most of the health care professionals who answered this survey were positive towards involving parents and delegating pain management to them. However, the results show that pain assessment by parents is underutilized, as are parent-targeted education on parent-delivered interventions and interprofessional education programs, which are therefore major areas for improvement. NICUs in low-income and low-tech settings were under-represented in our survey’s responses, making it difficult to draw conclusions in these contexts.

## Figures and Tables

**Table 1:** Description of responding units.

Level of Neonatal Care According to AAP ^1^ Guidelines	
I—well newborn nursery, n (%)	4 (1.3)
II—special care nursery, n (%)	38 (12.5)
III—neonatal intensive care unit, n (%)	151 (49.8)
IV—regional neonatal intensive care unit, n (%)	105 (34.7)
**Unit design**	
Single rooms, n (%)	60 (19.8)
Open bay, n (%)	102 (33.7)
Mixed design, n (%)	139 (45.9)
Number of beds, median (q1–q3)	10 (6–20)
Number of beds in which a parent can stay, median (q1–q3)	5 (1–13.5)
**World Bank income classification of the country**	
Low-income, n (%)	1 (0.3)
Lower–middle-income, n (%)	15 (5.0)
Upper–middle-income, n (%)	40 (13.2)
High-income, n (%)	247 (81.5)
**Sociodemographic index**	
Low SDI, n (%)	-
Low–middle SDI, n (%)	13 (4.3)
Middle SDI, n (%)	15 (6.3)
High SDI, n (%)	147 (48.5)

^1^ AAP = American Academy of Pediatrics, SDI = sociodemographic index, - indicates no answer in this category.

**Table 2:** Properties of the units belonging to the classes with different levels of parental participation in pain management.

Level of Parental Participation in Pain Management	Low (26.4%)	Moderate (49.8%)	High (23.8%)
**World Bank income** **classification of the country**			
Low or lower–middle	43.8%	37.5%	18.8%
Middle–high or high	25.4%	50.5%	24.0%
**World Bank** **region classification**			
East Asia and Pacific	60.0%	20.0%	20.0%
Europe and Central Asia	24.2%	50.4%	25.4%
South Asia	50.0%	50.0%	-
Latin America and the Caribbean	18.3%	50.0%	31.3%
North America	42.9%	47.6%	9.5%
Middle East and North Africa	16.7%	83.3%	-
Sub-Saharan Africa	38.5%	38.5%	23.1%
**Level of neonatal care** **according to AAP** ^1^ **guidelines**			
I—well newborn nursery	-	50.0%	50.0%
II—special care nursery	23.7%	65.8%	10.5%
III—neonatal intensive care unit	25.8%	48.3%	25.8%
IV—regional neonatal intensive care unit	28.6%	47.6%	23.5%
**Unit design**			
Single rooms	20.0%	56.7%	23.3%
Open bay	32.4%	47.1%	20.6%
Mixed design	23.7%	49.6%	26.6%

^1^ AAP = American Academy of Pediatrics, - indicates no answer in this category.

## Data Availability

The data presented in this study are available on request from the corresponding author. The data are not publicly available due to restrictions, e.g., privacy or ethical reasons.

## Supplementary Materials

The following supporting information can be downloaded at https://www.mdpi.com/article/10.3390/children11091105/s1. File S1: List of members in ESPR Special Interest Group for Neonatal Pain; File S2: List of members in PEARL research group; File S3: The survey.
