# Supplementary material for: Parents in Neonatal Pain Management—An International Survey of Parent-Delivered Interventions and Parental Pain Assessment
Source: Children (Basel). 2024 Sep 9;11(9):1105. doi: 10.3390/children11091105 (PMC11430199; doi:10.3390/children11091105)
Supplement: Supplementary file 1 [file children-11-01105-s001.zip › children-3140033-supplementary.pdf]

S1 Members of ESPR Neonatal Pain Special Interest Group

Members	Affiliation, City, Country
Abigail Kusi Amponsah	1. Department of Nursing, Faculty of Allied Health Sciences, College of Health Sciences, Kwame Nkrumah University of Science and Technology, Kumasi, Ghana.<br>2. Department of Nursing Science, Faculty of Medicine, University of Turku, Turku, Finland.
Agnes van den Hoogen	Department of Neonatology, Wilhelmina Children's Hospital, University Medical Centre Utrecht, Utrecht University, Utrecht, The Netherlands.
Alexandra Ullsten	1. Center for Clinical Research, Region Värmland, Karlstad, Sweden.<br>2. Faculty of Medicine and Health, School of Health Sciences, Örebro University, Örebro, Sweden.
Angela Amigoni	Pediatric Intensive Care Unit, Department of Woman's and Child's Health, University Hospital of Padova, Padova, Italy
Anna Axelin	Department of Nursing Science, University of Turku, Turku, Finland.
Anna-Kaija Palomaa	Department of Pediatrics and Adolescence, Oulu University Hospital, Oulu, Finland.
Anne Smits	1. Department of Development and Regeneration, KU Leuven, Leuven, Belgium<br>2. Neonatal Intensive Care Unit, University Hospitals Leuven, Leuven, Belgium
Aomesh Bhatt	Department of Paediatrics, University of Oxford, Oxford, United Kingdom.
Arild Rønnestad	1. Department of Neonatal Intensive Care, Oslo University Hospital, Oslo, Norway.<br>2. Institute of Clinical Medicine, University of Oslo, Oslo, Norway.
Beatrice Olsson Duse	Children and the Youth Clinic Eskilstuna Sweden.
Bente Johanne Vederhus	Department of Paediatric and Adolescent Medicine, Haukeland University Hospital, Bergen, Norway.
Bente Silnes Tandberg	1. Department of Paediatric and Adolescent Medicine, Drammen Hospital, Vestre Viken Hospital Trust, Drammen, Norway.<br>2. Lovisenberg Diaconal University College, Oslo, Norway.
Bert Joosten	1. Department of Anesthesiology and Pain Management, Maastricht University Medical Center + , Maastricht, The Netherlands<br>2. Department of Anesthesiology and Pain Management, Division of Translational Neuroscience, School of Mental Health and Neuroscience (MHeNs), University Maastricht, Maastricht, The Netherlands
Caroline Hartley	Department of Paediatrics, University of Oxford, Oxford, United Kingdom.
Catarina Sevivas	Neonatology Department, BCNatal, Barcelona Center for Maternal Fetal and Neonatal Medicine, Hospital Sant Joan de Déu and Hospital Clinic, University of Barcelona, Barcelona, Spain.<br>Neonatology Department, Clinical University Hospital of Santiago de Compostela, Santiago de Compostela, Spain.
Charalampos Kotidis	1. Department of Women's and Children's Health, University of Liverpool, Liverpool Health Partners, Liverpool, United Kingdom.<br>2. University of Liverpool, Liverpool Womens Hospital, Liverpool, United Kingdom.
Charles Christoph Roehr	1. National Perinatal Epidemiology Unit, Clinical Trials Unit, Oxford Population Health, Medical Sciences Division, University of Oxford, Oxford, United Kingdom.<br>2. Women and Children's, Neonatal Intensive Care Unit, Southmead Hospital, North Bristol NHS Trust, Westbury on Trym, Bristol, United Kingdom.
Christ-Jan van Ganzewinkel	Neonatal Intensive care Unit, Maxima Medical Centre, Veldhoven, The Netherlands.
Cristina Arribas	Neonatal Intensive Care Unit. Clínica Universidad de Navarra. Madrid. Spain.
Daniela Sofia Horta Machado	Neonatal Intensive Care Unit, Imperial College NHS TRUST, London, United Kingdom.
Daniëlla Roofthooft	Department of Pediatrics, Division of Neonatology, Erasmus MC Sophia Children's Hospital, Rotterdam, The Netherlands.
Eduardo Villamor	Division of Neonatology, MosaKids Children’s Hospital, Maastricht University Medical Center (MUMC+), Research Institute for Oncology and Reproduction (GROW), Maastricht University, 6202AZ Maastricht, The Netherlands.
Elisabeth Norman	1. Department of Neonatology, Skåne University Hospital, Lund, Sweden<br>2. Department of Paediatrics, Clinical Sciences, Lund University, Lund, Sweden.
Emma Olsson	1. Department of Paediatrics, Faculty of Medicine and Health, Örebro University, Örebro, Sweden<br>2. Faculty of Medicine and Health, School of Health Sciences, Örebro University, Örebro, Sweden
Eugene Dempsey	Department of Paediatrics and Child Health, Infant Centre, University College Cork, Cork, Ireland.
Evalotte Mörelius	1. Department of Health, Medicine and Caring Sciences, Linköping University, Linköping, Sweden.

	2. School of Nursing and Midwifery, Edith Cowan University, Perth, Australia.
Felipe Garrido	Neonatal Intensive Care Unit. Clínica Universidad de Navarra. Madrid. Spain
Flore Le Marechal	Department of Paediatric and Adolescent Medicine, Drammen Hospital, Vestre Viken Hospital Trust, Drammen, Norway.
Genny Raffaeli	Neonatal Intensive Care Unit. Fondazione IRCCS Ca' Granda Ospedale Maggiore Policlinico. Milan. Italy
Gerbrich van den Bosch	Department of Neonatal and Pediatric Intensive Care, Division of Neonatology, Erasmus University Medical Center - Sophia Children's Hospital, Rotterdam, The Netherlands
Giacomo Cavallaro	Neonatal Intensive Care Unit. Fondazione IRCCS Ca' Granda Ospedale Maggiore Policlinico. Milan. Italy
Guðrún Kristjánsdóttir	1. Faculty of Nursing and Midwifery, University of Iceland, Reykjavik, Iceland.<br>2. Children´s Hospital, Landspitali University Hospital, Reykjavik, Iceland
Hanna Ahl	Department of Neonatology, Skåne University Hospital, Lund, Sweden.
Hans Jørgen Stensvold	Department of Neonatal Intensive Care, Clinic of Paediatric and Adolescent Medicine, Oslo University Hospital, Oslo, Norway.
Helle Haslund-Thomsen	1. Clinical Nursing Research Unit, Aalborg University Hospital, Aalborg, Denmark.<br>2. Department of Paediatrics, Aalborg University Hospital, Department of Clinical Medicine, Aalborg University, Aalborg, Denmark
Janne Weiss	Department of Neonatology, The Juliane Marie Centre, Copenhagen University Hospital, Copenhagen, Denmark.
Jannicke Hanne Andresen	Department of Neonatology, Oslo University Hospital, Oslo, Norway.
Jean-Michel Roue	Department of Neonatal Medicine, University Hospital of Brest, Brest, France.
Joke Wielenga	IC Neonatology Emma Children's Hospital Amsterdam University Medical Center, Amsterdam, The Netherlands
Judith ten Barge	Department of Neonatal and Pediatric Intensive Care, Division of Neonatology, Erasmus MC –Sophia Children's Hospital, Rotterdam, the Netherlands
Karel Allegaert	1. Department of Development and Regeneration, KU Leuven, Leuven, Belgium.<br>2. Department of Hospital Pharmacy, Erasmus MC, Rotterdam, The Netherlands<br>3. Department of Pharmaceutical and Pharmacological Sciences, KU Leuven, Leuven, Belgium
Katrin Klebermass-Schrehof	Division of Neonatology, Pediatric Intensive Care and Neuropediatrics, Department of Pediatrics and Adolescent Medicine, Comprehensive Center for Pediatrics, Medical University Vienna, Austria
Laila Kristoffersen	1. Department of Neonatology, St Olavs Hospital Trondheim University Hospital, Trondheim, Norway.<br>2. Department of Public Health and Nursing, Norwegian University of Science and Technology, Trondheim, Norway.
Laura Moschino	Neonatal Intensive Care Unit, Padova University Hospital, Padova, Italy
Lene Lyngstad	Department of Paediatric and Adolescent Medicine, Drammen Hospital, Vestre Viken Hospital Trust, Drammen, Norway.
Liam Mahoney	1. University Hospitals Bristol and Weston NHS Foundation Trust, Bristol, United Kingdom.<br>2. Regional Neonatal Intensive Care Unit St. Michael's Hospital, Bristol, United Kingdom.
Luke Baxter	Department of Paediatrics, University of Oxford, Oxford, United Kingdom.
Magdalena Panek	Department of Maternal and Child Health, Faculty of Health Sciences, Jagiellonian University Medical College, Krakow, Poland.
Manon Tauzin	Neonatal Intensive Care Unit, Centre Hospitalier Intercommunal de Créteil, Créteil, France.
Maria Gradin	Department of Pediatrics, Faculty of Medicine and Health, Örebro University, Örebro, Sweden.
Mariaana Mäki-Asiala	1. Research Unit of Health Sciences and Technology, Faculty of Medicine, University of Oulu, Oulu, Finland.<br>2. Medical Research Center Oulu, Oulu University Hospital, University of Oulu, Oulu, Finland.
Marsha Campbell-Yeo	School of Nursing, Faculty of Health, Departments of Pediatrics, Psychology and Neuroscience, Dalhousie University and IWK Health, Halifax, Nova Scotia, Canada
Marta Camprubí Camprubí	1. Department of Neonatology, Hospital Sant Joan de Déu, Barcelona, Spain.<br>2. Cardiovascular Research Group, Sant Joan de Déu Research Institute, Barcelona, Spain.<br>3. BCNatal-Barcelona Center for Maternal Fetal and Neonatal Medicine, Hospital Sant Joan de Déu-Hospital Clinic, University of Barcelona, Barcelona, Spain.
Martina Carlsen Misic	4. Department of Pediatrics, Faculty of Medicine and Health, Örebro University, Örebro, Sweden.<br>5. Faculty of Medicine and Health, School of Health Sciences, Örebro University, Örebro, Sweden.

Mathilde Baudat	1. Department of Anesthesiology and Pain Management, Maastricht University Medical Centre+, Maastricht, The Netherlands<br>2. Department of Translational Neuroscience, School of Mental Health and Neuroscience, Maastricht University, Maastricht, The Netherlands.
Mats Eriksson	School of Health Sciences, Faculty of Medicine and Health, Örebro University, Örebro, Sweden
Máximo Vento	Neonatal Research Group at the Health Research Institute La Fe (IISLAFE), Valencia, Spain.
Monica Riaza Gomez	Unidad de Neonatología, Departamento de Pediatría, HM Hospitales, Madrid, Spain
Monique van Dijk	1. Department of Neonatal and Pediatric Intensive Care, Division of Pediatric Intensive Care, Erasmus MC-Sophia Children's Hospital, Erasmus University Medical Center Rotterdam, Rotterdam, The Netherlands.<br>2. Department of Internal Medicine, Division of Nursing Science, Erasmus MC, Erasmus University Medical Center Rotterdam, Rotterdam, The Netherlands.
Naomi Meesters	Department of Neonatal and Pediatric Intensive Care, Division of Neonatology, Erasmus MC –Sophia Children's Hospital, Rotterdam, the Netherlands
Natalia Toumbourou	Neonatal Intensive Care Unit, Western Health Hospital, Melbourne, Australia
Nunzia Decembrino	Neonatal Intensive Care Unit, AOU Policlinico G. Rodolico San Marco, Catania, Italy
Paola Lago	Neonatal Intensive Care Unit, Department of Critical Care, Cà Foncello Regional Hospital, Treviso, Italy.
Pia Lundqvist	Department of Health Sciences, Lund University, Lund, Sweden.
Randi Dovland Andersen	1. Department of Research, Telemark Hospital Trust, Skien, Norway<br>2. Research Centre for Habilitation and Rehabilitation Models & Services (CHARM), The Faculty of Medicine, University of Oslo, Oslo, Norway
Rebeccah Slater	Department of Paediatrics, University of Oxford, Oxford, United Kingdom.
Ricardo Carbajal	1. Pediatric Emergency Department, APHP - Hôpital Armand Trousseau, Sorbonne Université, Paris, France<br>2. INSERM UMR 1153, Paris, France
Rikke Louise Stenkjær	Department of Intensive Care for Infants and Toddlers, Copenhagen University Hospital Rigshospitalet, Copenhagen, Denmark.
Robert B. Flint	1. Department of Hospital Pharmacy, Erasmus University Medical Center, Rotterdam, The Netherlands<br>2. Department of Pediatrics, Division of Neonatology, Erasmus University Medical Center - Sophia Children's Hospital, Rotterdam, The Netherlands
Samir El Abdouni	Department of Hospital Pharmacy, Erasmus University Medical Center, Rotterdam, The Netherlands
Scott Montgomery	1. Clinical Epidemiology and Biostatistics, Department of Medical Sciences, Faculty of Medicine and Health, Örebro University, Sweden.<br>2. Clinical Epidemiology Division, Department of Medicine, Solna, Karolinska Institute, Stockholm, Sweden.<br>3. Department of Epidemiology and Public Health, University College London, London, United Kingdom.
Serdar Beken	Department of Pediatrics, Section of Neonatology, Acibadem Mehmet Ali Aydinlar University, School of Medicine, İstanbul, Turkey.
Sezin Ünal	Division of Neonatology, Baskent University Faculty of Medicine, Ankara, Turkey.
Shalini Ojha	1. Centre for Perinatal Research, School of Medicine, University of Nottingham, Nottingham, United Kingdom.<br>2. Neonatal Unit, University Hospitals of Derby and Burton NHS Trust, Derby, United Kingdom.
Shellie Robinson	Department of Paediatrics, University of Oxford, Oxford, United Kingdom.
Sigríður María Atladóttir	Faculty of Nursing, University of Iceland, Reykjavik, Iceland.<br>Neonatal Intensive Care Unit, Lanspitali University Children's Hospital, Reykjavik, Iceland.
Sinno HP Simons	Department of Pediatric and Neonatal Intensive Care, division of Neonatology, Erasmus University Medical Center - Sophia Children's Hospital, Rotterdam, The Netherlands
Sofie Pirlotte	Neonatal Intensive Care Unit, University UZ Brussel, Brussels, Belgium
Solfrid Steinnes	Department of Paediatric and Adolescent Medicine Neonatal Intensive Care Unit Drammen Hospital Vestre Viken Hospital Trust Drammen Norway.
Swantje Völler	Division of Systems Pharmacology and Pharmacy, Leiden Academic Centre for Drug Research, Leiden University, Leiden, The Netherlands.

Tarja Pölkki	1. Research Unit of Health Sciences and Technology, University of Oulu, Oulu, Finland<br>2. Medical Research Center Oulu, Oulu University Hospital and University of Oulu, Oulu, Finland
Tiina Ukkonen	Department of Pediatrics, Oulu University Hospital, Oulu, Finland.
Tom Stiris	1. Department of Neonatal Intensive Care, Oslo University Hospital, Oslo, Norway.<br>2. Institute of Clinical Medicine, University of Oslo, Oslo, Norway.
Xavier Durrmeyer	Neonatal Intensive Care Unit, CHI Créteil, Créteil, France.

S2 Members of Pain in Early Life (PEARL) Research Group

Hanna Ahl, Department of Neonatology, Skåne University Hospital, Lund, Sweden

Abigail Kusi Amponsah, Department of Nursing, Faculty of Allied Health Sciences, College of Health Sciences, Kwame Nkrumah University of Science and Technology, Kumasi, Ghana.

Randi Dovland Andersen, Department of Research, Telemark Hospital Trust, Skien, Norway

Sigríður María Atladóttir, Faculty of Nursing, University of Iceland, Reykjavik, Iceland

Anna Axelin, Department of Nursing Science, University of Turku, Turku, Finland

Marsha Campbell-Yeo, School of Nursing, Faculty of Health, Departments of Pediatrics, Psychology and Neuroscience, Dalhousie University and IWK Health, Halifax, Nova Scotia, Canada

Martina Carlsen-Misic, School of Health Sciences, Faculty of Medicine, Örebro University and Örebro University Hospital, Örebro, Sweden

Mats Eriksson, School of Health Sciences, Faculty of Medicine, Örebro University, Örebro, Sweden

Maria Gradin, Örebro University Hospital, Örebro, Sweden

Helle Haslund-Thomsen, Department of Paediatrics, Aalborg University Hospital, Department of Clinical Medicine, Aalborg University, Aalborg, Denmark

Guðrún Kristjánsdóttir, Faculty of Nursing and Midwifery, University of Iceland, Reykjavik, Iceland

Laila Kristoffersen, Department of Neonatology, St Olavs Hospital Trondheim University Hospital, Trondheim, Norway

Pia Lundqvist, Department of Health Sciences, Lund University, Lund, Sweden.

Lene Tandle Lyngstad, Department of Paediatric and Adolescent Medicine, Drammen Hospital, Vestre Viken Hospital Trust, Drammen, Norway.

Scott Montgomery, Clinical Epidemiology and Biostatistics, Department of Medical Sciences, Faculty of Medicine and Health, Örebro University, Sweden

Mariaana Mäki-Asiala, 1. Research Unit of Health Sciences and Technology, Faculty of Medicine, University of Oulu, Oulu, Finland

Evalotte Mörelius, Department of Health, Medicine and Caring Sciences, Linköping University, Linköping, Sweden

Beatrice Olsson Duse, Children and the Youth Clinic Eskilstuna Sweden

Emma Olsson, School of Health Sciences, Faculty of Medicine, Örebro University and Örebro University Hospital, Örebro, Sweden

Anna-Kaija Palomaa, Department of Pediatrics and Adolescence, Oulu University Hospital, Oulu, Finland

Magdalena Panek, Department of Maternal and Child Health, Faculty of Health Sciences, Jagiellonian University Medical College, Krakow, Poland

Tarja Pölkki, Medical Research Center Oulu, Oulu University Hospital and University of Oulu, Oulu, Finland

Solfreid Steinnes, Department of Paediatric and Adolescent Medicine Neonatal Intensive Care Unit Drammen Hospital Vestre Viken Hospital Trust Drammen Norway.

Rikke Louise Stenkjaer, Department of Intensive Care for Infants and Toddlers, Copenhagen University Hospital Rigshospitalet, Copenhagen, Denmark

Alexandra Ullsten, Center for Clinical Research, Region Värmland, Karlstad, and Faculty of Medicine and Health, School of Health Sciences, Örebro University, Örebro, Sweden

Bente Vederhus, Department of Paediatric and Adolescent Medicine, Haukeland University Hospital, Bergen, Norway

Janne Weis, Department of Neonatology, The Juliane Marie Centre, Copenhagen University Hospital, Copenhagen, Denmark.

S3 The Survey

Welcome to a survey about the role of parents in neonatal pain management

The survey is performed by a working group within the Special Interest Group on Neonatal Pain within European Society of Pediatric Research (ESPR Neonatal Pain SIG) and the Pain in Early Life (PEARL) network. It is distributed through a number of professional and scientific networks around the globe.

The survey explores the conditions on a unit level, i.e. not of any indivudual. The idea is that each neonatal unit responds once to the survey, so please ask the person with the most knowledge about pain management to deliver the answers. We will ask you about your hospital and name/number of the NICU. This is to be able to sort out duplicate answers from the same unit. Your answers will be treated with confidentiality and the results will be reported on a group level where no individual unit can be identified.

Participation is voluntary but it is our hope that you will take the time (approximately 10-15 minutes) to answer the survey.

If you have any questions about the survey, please contact professor Mats Eriksson: mats.h.eriksson@oru.se or Alexandra Ullsten, PhD: alexandra.ullsten@oru.se.

Please klick Next page to continue to the survey.

Country

City

Name of hospital

Name / number of the unit (i.e. how is the unit identified at the hospital?)

Profession of the person responding to the survey

- ☐ Neonatologist
- ☐ Pediatrician
- ☐ Other physiscian, medical doctor
- ☐ Nurse, registered nurse
- ☐ Other

If other, please specify

Position / role of the person answering the survey

- ☐ Person with medical responsibility for the NICU, e.g. chief physician
- ☐ Person with responsibility for nursing, e.g. chief nurse
- ☐ Other

If other, please specify

Information about the unit and possibilities for parents to be with their newborn infant

What is the design of the unit?

- ☐ Single rooms
- ☐ Open bay
- ☐ Mixed

Number of infant beds in single rooms (if existing)

[ ]

Number of infant beds in open bay areas (if existing)

[ ]

Level of neonatal care according to AAP guidelines (1999). If more than one level, select the highest.

- ☐ Level I - well newborn nursery
- ☐ Level II - special care nursery
- ☐ Level III - NICU
- ☐ Level IV - regional NICU

Number of beds in the unit where parents can stay overnight.

[ ]

In your country / region, what is the number of parental leave days (social benefit) that parents can use to stay at the hospital with their sick or preterm newborn infant? If several options, please give the answer that is most typical for your unit.

[ ]

Here you can comment on the questions in this section.

[ ]

Guidelines or protocols for neonatal pain management

Does the unit have pain management guidelines / protocols? (more than one answer is possible)

☐ No guidelines

☐ Yes, locally developed guidelines

☐ Yes, regional or national guidelines

In your opinion, how well does the staff at the unit follow the guidelines / protocols? (skip if the above answer is No)

☐ Never

☐ Seldom / not very well

☐ About half of the time / acceptable

☐ Most of the time / very well Always

☐

Is the role of parents in pain management mentioned in the guidelines / protocols?

☐ Not mentioned

☐ Mentioned as an option Recommended

☐

Here you can comment on the questions in this section.

Parent-delivered interventions

Do parents provide pain relief in your unit? (e.g. by using skin-to-skin contact, breastfeeding, singing etc.)

- ☐ Never
- ☐ Very seldom
- ☐ Occasionally
- ☐ On a regular basis
- ☐ Always

Which of the following parent-delivered interventions are mentioned in your guidelines / protocols?

- ☐ Breastfeeding
- ☐ Skin-to-skin contact/kangaroo mother care
- ☐ Live singing / humming
- ☐ Facilitated tucking
- ☐ Clothed holding (not skin-to-skin)
- ☐ Containment / swaddling
- ☐ Touch
- ☐ Massage
- ☐ Non-nutritive sucking
- ☐ Sensorial saturation
- ☐ Combined interventions (e.g. skin-to-skin contact and breastfeeding. Please specify below)
- ☐ Other

If other, please specify

If combined parent-delivered interventions are mentioned or recommended in the guidelines / protocols, please specify what combined interventions are recommended.

Which of the following parent-delivered interventions are actually performed by parents in your unit?

	Never	Very seldom	Occasionall-y	On a regular basis	Always
Breastfeeding	☐	☐	☐	☐	☐
Skin-to-skin contact/ kangaroo mother care	☐	☐	☐	☐	☐
Live singing / humming	☐	☐	☐	☐	☐
Facilitated tucking	☐	☐	☐	☐	☐
Clothed holding (not skin-to-skin)	☐	☐	☐	☐	☐
Containment / swaddling	☐	☐	☐	☐	☐
Touch	☐	☐	☐	☐	☐
Massage	☐	☐	☐	☐	☐
Non-nutritive sucking	☐	☐	☐	☐	☐
Sensorial saturation	☐	☐	☐	☐	☐
Combined interventions (please specify below)	☐	☐	☐	☐	☐
Other	☐	☐	☐	☐	☐

If other, please specify

If combined parent-delivered interventions are performed in the unit, please specify what interventions are commonly combined at your unit.

If parents in the unit are asked to perform parent-delivered pain-relieving interventions, in your opinion, how do they react / respond?

- ☐ Very negative
- ☐ Negative
- ☐ Neutral
- ☐ Positive
- ☐ Very positive

Here you can comment on the questions in this section.

Pain assessment

Is pain assessment by parents mentioned or recommended in your guidelines / protocols?

☐ Yes No

☐

Is pain assessment performed by parents?

☐ Never

☐ Very seldom

☐ Occasionally

☐ On a regular basis

☐ Always

If pain assessment is performed by parents, please describe in which situations. Is a pain scale used, and in that case which one?

Here you can comment on the questions in this section.

Attitudes of the staff

In your own opinion, what is the opinion of the (majority of the) registered nurses at the unit towards the use of parent-delivered interventions?

- ☐ Very negative
- ☐ Negative
- ☐ Neutral
- ☐ Positive
- ☐ Very positive

In your own opinion, what is the opinion of the (majority of the) physisicans (including neonatologists, pediatricians and consultants) at the unit towards the use of parent-delivered interventions?

- ☐ Very negative
- ☐ Negative
- ☐ Neutral
- ☐ Positive
- ☐ Very positive

In your own opinion, what is the opinion of the (majority of the) nurse assistants (meaning other staff involved in direct care, except registerd nurses) at the unit towards the use of parent-delivered interventions?

- ☐ Very negative
- ☐ Negative
- ☐ Neutral
- ☐ Positive
- ☐ Very positive

Here you can comment on the questions in this section.

Thank you for answering the survey. When you are ready, please click submit.
